# Second Prenatal Diagnosis of Bosch–Boonstra–Schaaf Optic Atrophy Syndrome in a Fetus With a 5q14.3q15 Deletion: A Case Report and Review of the Literature

**DOI:** 10.1002/ccr3.72581

**Published:** 2026-04-19

**Authors:** Ying Hao, Qingfa Huang, Yong Xu, Xingping Li, Peining Li, Weiqing Wu, Bo Wu, Wenlan Liu

**Affiliations:** ^1^ Medical Genetic Center, Shenzhen Maternity and Child Healthcare Hospital, Women and Children's Medical Center Southern Medical University Shenzhen Guangdong China; ^2^ Department of Genetics Yale University School of Medicine New Haven Connecticut USA; ^3^ Shenzhen Key Laboratory of Maternal and Child Health and Diseases China

**Keywords:** 5q14.3q15 deletion, Bosch–Boonstra–Schaaf optic atrophy syndrome (BBSOAS), cell‐free DNA screening, molecular genetics, *NR2F1* gene, prenatal diagnosis

## Abstract

This case demonstrates the value of cell‐free DNA (cfDNA) screening for detecting subchromosomal microdeletions in fetuses with non‐specific prenatal screening abnormalities and no overt structural malformations on ultrasound; CMA and karyotyping confirmation and integrated genetic counseling are essential for diagnosing 5q14.3q15 deletion‐related BBSOAS and guiding parental decision‐making.

## 1 Introduction

Bosch–Boonstra–Schaaf optic atrophy syndrome (BBSOAS, OMIM# 615722) is a rare autosomal dominant disorder characterized by a spectrum of clinical features of cerebral visual impairment, developmental delay, and intellectual disability [1]. In 2014, Bosch et al. [1] described the first six individuals with heterozygous missense variants or deletions in the *NR2F1* gene; all showed optic nerve abnormalities and intellectual impairment. Accumulated case series provided evidence for genotype–phenotype correlations by heterozygous loss‐of‐function variants in the *NR2F1* gene and by haploinsufficiency due to a deletion encompassing the gene [2, 3]. The *NR2F1* gene, also known as *COUP‐TFI*, is mapped at 5q15 and encodes a protein that acts as a nuclear receptor and transcriptional regulator [4]. The prevalence of BBSOAS is estimated to be about 1/100,000–1/250,000 people worldwide [5].

To date, approximately 100 newborn and pediatric patients have been reported, but only one fetus was diagnosed prenatally [5, 6, 7]. This substantial discrepancy highlights a significant gap in the current understanding of the disease during the prenatal stage, with its prenatal phenotypic spectrum, natural history, and optimal prenatal diagnostic strategies yet to be elucidated. In view of this, each newly confirmed prenatal case is particularly valuable, not only enriching our knowledge of the prenatal manifestations of BBSOAS but also providing critical information for optimizing genetic counseling and perinatal management.

Here, we report one fetus with BBSOAS caused by a deletion of 5q14.3q15 encompassing the *NR2F1* gene, representing the second prenatal case described in the literature. By detailing the prenatal ultrasound findings, cell‐free DNA (cfDNA) screening results, molecular diagnostic process, and family validation for this case, we aim to (1) further expand the prenatal phenotypic spectrum of BBSOAS; (2) highlight the clinical utility and challenges of cfDNA screening in detecting such microdeletion syndromes; and (3) provide practical references for clinicians and genetic counselors regarding diagnostic strategies, prognostic counseling, and perinatal management when encountering similar cases.

## 2 Case Presentation/Examination

A 32‐year‐old pregnant woman, gravida 2, para 1, was found to have a nuchal translucency (NT) measurement of 0.26 cm and a high‐risk serum screening result of 1/20 for trisomy 21 during first‐trimester screening. Subsequent cfDNA screening excluded high risk for chromosomal aneuploidies but unexpectedly revealed a 7.64‐Mb deletion at chromosome 5q14.3q15. This pregnancy was conceived naturally. The couple were nonconsanguineous, healthy without any histories of drugs, infections, or genetic diseases. The amniocentesis was performed at 17 weeks of gestation for the purpose of prenatal diagnosis to confirm the fetal karyotype and to verify the 5q14.3q15 deletion detected by cfDNA screening after fully written consent. Conventional G‐banded karyotyping and chromosomal microarray analysis (CMA) using a single nucleotide polymorphism (SNP) array platform by Affymetrix CytoScan 750 K arrays were performed as previously described [8].

**FIGURE 1:**
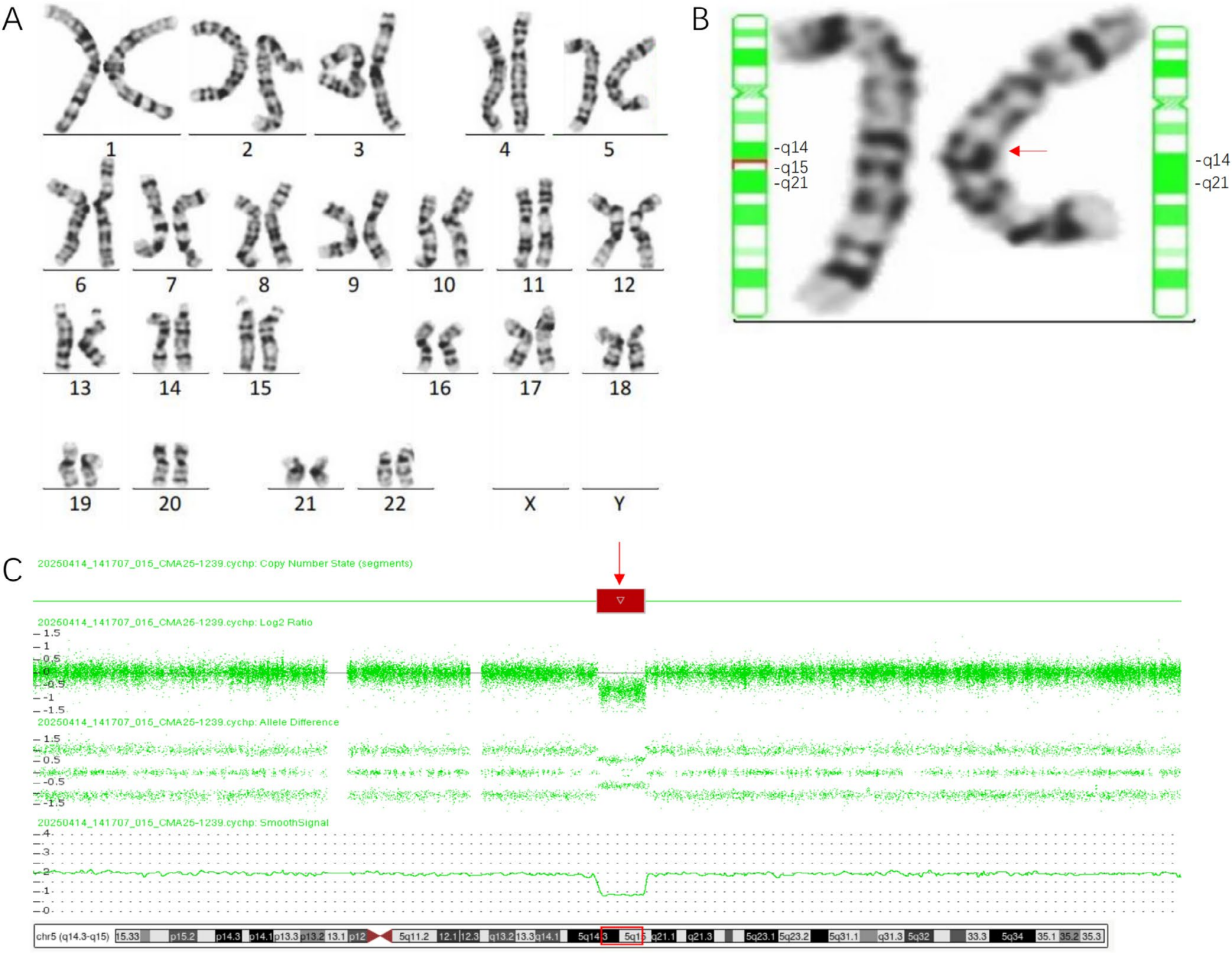
(A) Karyotype showed 46,XN,del(5)(q14.3q15). (B) Chromosome 5 and the deletion fragment of chromosome 5 (red arrow). (C) CMA result showed a 7.46‐Mb deletion on 5q14.3q15 (89009930_96465325) (GRCh37/hg19). The region of 5q14.3q15 is boxed in red.

CMA on the DNA extracted from uncultured amniocytes revealed the result of arr[GRCh37] 5q14.3q15(89009930_96465325)x1 with a 7.46‐Mb deletion encompassing 22 Online Mendelian Inheritance in Man (OMIM) genes. The minor discrepancy in size between the cfDNA and CMA results is due to differences in the sample source and testing principles as well as the depth of coverage of the two methods. Simultaneous karyotyping on the cultured amniocytes revealed the result of 46,XN,del(5)(q14.3q15) (Figure 1). The absence of fetal sex information in the prenatal diagnosis report is due to legal prohibitions against sex determination for non‐medical reasons. The deletion fragment at 5q14.3q15 involved the whole *NR2F1* gene, and haploinsufficiency of this gene was associated with BBSOAS. The karyotyping of the couple showed normal results and indicated a de novo deletion in the fetus.

## 3 Methods (Differential Diagnosis, Investigations and Treatment Related to the Current Case)

The primary differential diagnosis for the fetal 5q14.3q15 deletion identified by cfDNA screening and confirmatory testing was BBSOAS, caused by *NR2F1* haploinsufficiency—the only well‐characterized monogenic disorder associated with deletions in this chromosomal region. Additional differential considerations for 5q14.3q15 deletions included other rare neurodevelopmental disorders caused by haploinsufficiency of other OMIM genes within the deletion fragment (22 total OMIM genes in this case). Key differential diagnoses were as follows.

*MEF2C* haploinsufficiency syndrome (OMIM# 613443): Caused by deletion/mutation of *MEF2C* (not included in the 5q14.3q15 deletion in this case), characterized by intellectual disability, epilepsy, autism, and brain malformations excluded via CMA gene content analysis.

Isolated 5q14.3q15 microdeletion syndrome: A rare non‐syndromic neurodevelopmental disorder with overlapping features (developmental delay) but no optic atrophy; distinguished from BBSOAS by the inclusion of *NR2F1* in the deletion (the defining feature of BBSOAS).

Other syndromic microdeletion disorders: 5q chromosomal deletions outside the 5q14.3q21.3 region (e.g., 5q31 deletion syndrome) were excluded via precise CMA mapping of the deletion breakpoints.

All investigative procedures were performed in accordance with clinical prenatal diagnosis guidelines of the Chinese Medical Association and the American College of Medical Genetics and Genomics (ACMG). No in utero therapeutic interventions were indicated for this case, as BBSOAS is a genetic neurodevelopmental disorder with no curative prenatal or postnatal treatment available.

## 4 Conclusion and Results (Outcome and Follow‐Up Related to the Current Case)

Even though prenatal ultrasound at 21 weeks of gestation did not detect any fetal structural abnormalities, the couple chose to terminate the pregnancy at 22 weeks of gestation after receiving thorough genetic counseling and being well informed of fetal possible severe adverse outcomes after labor. This process was approved by Shenzhen Maternity and Child Healthcare Hospital Ethics Committee.

In summary, this case was the second prenatal case with BBSOAS caused by a deletion of 5q14.3q15. The prenatal ultrasound findings of BBSOAS are non‐specific and may include bilateral mild ventriculomegaly, intracranial cysts, increased NT, polyhydramnios, and soft ultrasound markers. All the 17 reported cases of BBSOAS caused by deletions at 5q14.3q21.3 contained the *NR2F1* gene, and approximately 82% of deletions are de novo and 18% are inherited. This study reported for the first time the detection of a BBSOAS fetus presenting with increased NT, expanding the prenatal phenotypic spectrum of the disease. It also represents the first reported case of a 5q14.3q15 deletion identified through cfDNA screening and confirmed by prenatal diagnosis and underscores the importance of cfDNA screening, particularly for fetuses without severe structural abnormalities on ultrasound. For cases with positive screening results, prenatal diagnosis should be performed for confirmation. Given the uncertainty in predicting prenatal phenotypes of BBSOAS, molecular diagnostic techniques are crucial for the prenatal diagnosis of affected fetuses. For clinicians and genetic counselors working on prenatal cases, information from cfDNA screening, ultrasound examination, invasive prenatal testing for cytogenetic and molecular genetics, family history, and parental testing should be provided and integrated to facilitate informed counseling and decision‐making for these families.

## 5 Discussion

**FIGURE 2:**
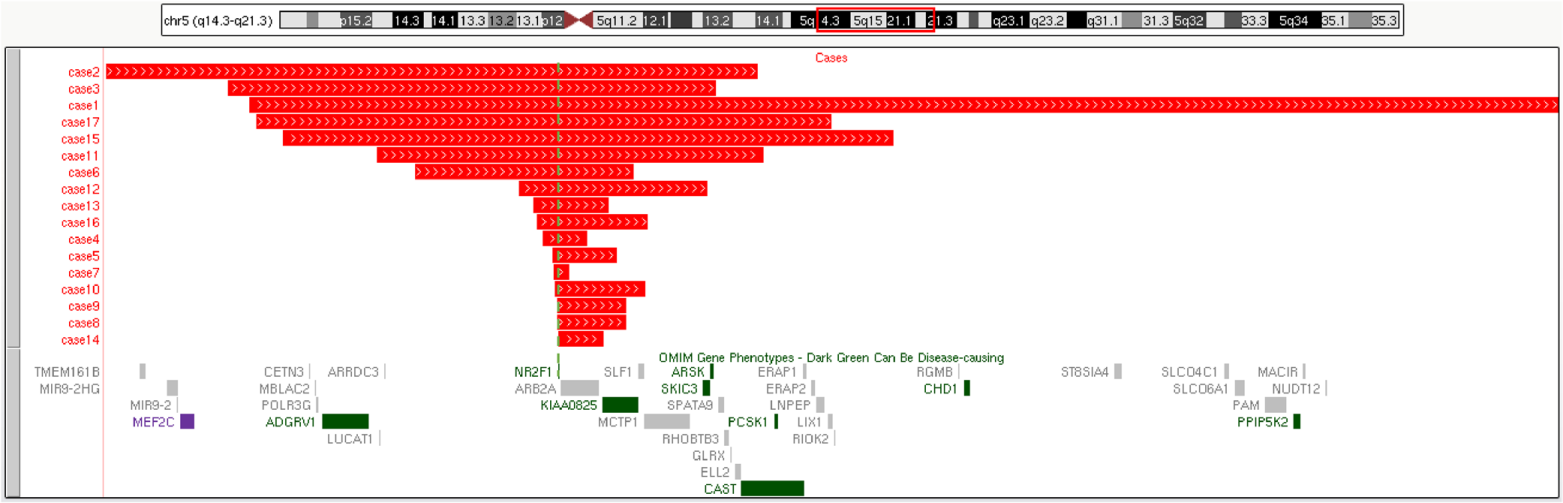
Cytogenomic mapping and genotype–phenotype correlations for candidate genes of 5q14.3q21.3 [UCSC Genome Browser on Human (GRCh37/hg19)]. A cytogenomic map for sizes of deletions and candidate genes of 5q14.3q21.3. The upper panel shows chromosome 5 with the band location information. The region of 5q14.3q21.3 is boxed in red. The middle panel shows the size and location of the 17 deletions. The lower panel shows OMIM genes in 5q14.3q21.3. The green dashed line indicates the location of the *NR2F1* gene.

We systematically searched all literature on *NR2F1* gene deletions in the PubMed database using keywords including *NR2F1* or BBSOAS. Cases that were confirmed by genetic testing and had clearly defined deletion breakpoints were included (Table 1, Figure 2) [1, 2, 3, 7, 9, 10, 11, 12]. All 17 cases carried a deletion ranging from 198 Kb to 17 Mb at 5q14.3q21.3. With the exception for the two fetuses, the ages of these *NR2F1* deletion patients ranged from 2 years old to 37 years old. Of the 11 cases with follow‐up parental analysis, nine (82%) had de novo deletions and two (18%) were inherited from a maternal or paternal carrier. All cases involved the *NR2F1* gene, which has a haploinsufficiency score of three. In addition to this gene, case 2 [9] also involved the haploinsufficiency gene *MEF2C*. The case was a 5‐year‐old girl whose clinical manifestations included severe developmental delay, absence of speech, epilepsy, and minor dysmorphic features. *MEF2C* haploinsufficiency syndrome (OMIM# 613443) is characterized by intellectual disability, epilepsy, autism, abnormal movements, brain abnormalities, and some dysmorphic features. These clinical features are similar to the clinical manifestations of BBSOAS, except for the visual impairment specific to BBSOAS, which includes optic nerve atrophy, optic nerve hypoplasia, and cortical visual impairment. Therefore, it cannot be determined whether the causative gene in this child is *NR2F1*, *MEF2C*, or a combination of both genes.

**TABLE 1:** BBSOAS caused by *NR2F1* gene deletion in 17 cases.

case	Age (years)	Chromosome mapping	Copy number variation (GRCh37)	Size (Mb)	Number of OMIM genes^a^	Inheritance	References
1	7	5q14.3q21.3	chr5:88909319‐105901597	16.992	33	de novo	Cardoso et al. [9], Patient 1
2	5	5q14.3q15	chr5:87050542‐95512884	8.462	21	de novo	Cardoso et al. [9], Patient 2
3	5	5q14.3q15	chr5:88623732‐94960785	6.337	13	de novo	Cardoso et al. [9], Patient 3
4	8.3	5q15	chr5:92717119‐93298594	0.581	2	de novo	Al‐Kateb et al. [10]
5	24	5q15	chr5:92845157‐93679748	0.835	3	NA	Bosch et al. [1], Patient 4
6	4	5q14.3q15	chr5:91064110‐93896378	2.832	3	de novo	Bosch et al. [1], Patient 5
7	8	5q15	chr5:92856299‐93054636	0.198	2	de novo	Chen et al. [2], Patient 16
8	35	5q15	chr5:92910393‐93806933	0.897	3	NA	Chen et al. [2], Patient 17
9	2	5q15	chr5:92910393‐93806933	0.897	3	Paternal	Chen et al. [2], Patient 18
10	37	5q15	chr5:92878375‐94046216	1.168	5	NA	Chen et al. [2], Patient 19
11	6	5q14.3q15	chr5:90566268‐95580992	5.015	13	NA	Chen et al. [2], Patient 20
12	16.6	5q15	chr5:92414689‐94864863	2.450	6	Maternal	Rech et al. [3], Patient 25
13	3.6	5q15	chr5:92594997‐93569402	0.974	3	NA	Rech et al. [3], Patient 26
14	6	5q15	chr5:92914091‐93513068	0.599	3	de novo	Jurkute, Bertacchi et al. [11], NR2F1_22
15	Fetus	5q14.3q15	chr5:89340000‐97280000	7.940	24	NA	Sun et al. [7]
16	13	5q15	chr5:92639433‐94084928	1.445	5	de novo	Hayashi et al. [12]
17	Fetus	5q14.3q15	chr5:89009930‐96465325	7.455	23	de novo	Our study

Abbreviations: BBSOAS, Bosch–Boonstra–Schaaf optic atrophy syndrome; NA, not available; OMIM, Online Mendelian Inheritance in Man.

^a^ The number of OMIM genes is determined in the ClinGen database.

The first fetal case with BBSOAS was reported in 2022 [7]. The fetus presented with bilateral mild ventriculomegaly and intracranial cyst by imaging examinations in the late second and early third trimester. Copy number variation sequencing (CNV‐Seq) combined with karyotyping confirmed a novel 7.94 Mb deletion on 5q14.3q15 (89340000_97280000), involving the whole *NR2F1* gene. The pregnancy was terminated in the end. The authors suggested that fetal intracranial phenotype abnormality may originate from the deletion of the *NR2F1* gene. Postnatal neuroimaging of BBSOAS patients also revealed ventricular enlargement and asymmetry, and BBSOAS mouse models demonstrate ventricular enlargement due to neocortical malformations during neurogenesis [13].

Here, we reported the second prenatal case with BBSOAS caused by a deletion of 5q14.3q15. The size of the deletion is similar to that of the first fetal case, and both involved the whole *NR2F1* gene. However, the fetal case we reported did not show structural abnormalities on ultrasound except for the increased NT. It was referred for prenatal diagnosis merely due to abnormal ultrasound soft markers, high‐risk results in serum screening, and abnormal cfDNA screening findings. Therefore, this clinico‐cytogenomic association of hydrocephalus with deletions of 5q14.3q15 still remains to be verified from more prenatal cases or related animal research results. It is known that increased NT is closely related to adverse pregnancy outcomes such as fetal chromosomal abnormalities, fetal malformations, and genetic syndromes and has become an important indicator for prenatal screening [14]. This study reported for the first time the detection of BBSOAS in a fetus with increased NT, expanding the prenatal phenotypic spectrum. In addition to efficient screening for trisomy 21, 18, and 13, cfDNA screening also has good detection efficiency for sex chromosome abnormalities and even CNV. The screening efficiency of cfDNA screening for pathogenic CNVs with fragments larger than 6 Mb can reach 83.0%–90.9% [15, 16]. This is the first reported case of a 5q14.3q15 deletion confirmed by prenatal diagnosis after being identified through cfDNA screening.

In addition to the two fetal cases, another 9‐year‐old child with BBSOAS caused by a frameshift mutation in the *NR2F1* gene had polyhydramnios during the fetal period [17]. To the best of our knowledge, these are the prenatal manifestations of BBSOAS cases reported so far. It can be seen from this that there are no specific prenatal ultrasonic manifestations of BBSOAS. Hence, molecular diagnostic techniques are critical for prenatal diagnosis on fetus with BBSOAS.

## Author Contributions

**Ying Hao:** conceptualization, writing – original draft. **Qingfa Huang:** data curation, investigation. **Yong Xu:** data curation, investigation. **Xingping Li:** validation. **Peining Li:** conceptualization. **Weiqing Wu:** supervision. **Bo Wu:** funding acquisition, project administration. **Wenlan Liu:** funding acquisition, project administration, writing – review and editing.

## Funding

This work was supported by grants from Shenzhen Health Economics Association (202330); Sanming Project of Medicine in Shenzhen Municipality (SZSM202311005); and Shenzhen Key Laboratory of Maternal and Child Health and Diseases (ZDSYS20230626091559006).

## Consent

We obtained written informed consent from the parents of the patient regarding the publication of this case.

## Conflicts of Interest

The authors declare no conflicts of interest.

## Data Availability

The data that support the findings of this study are available from the corresponding author upon reasonable request.
